# Decreased Ovarian Reserve Predicts Inexplicability of Recurrent Miscarriage? A Retrospective Analysis

**DOI:** 10.1371/journal.pone.0161606

**Published:** 2016-09-14

**Authors:** Sophie Pils, Regina Promberger, Stephanie Springer, Elmar Joura, Johannes Ott

**Affiliations:** 1 Department of Gynecological Endocrinology and Reproductive Medicine, Medical University of Vienna, Vienna, Austria; 2 Department of Obstetrics and Gynecology, Saint John of God Hospital Eisenstadt, Burgenland, Austria; University Hospital of Münster, GERMANY

## Abstract

**Objective:**

To evaluate anti-Mullerian hormone, basal follicle stimulating hormone, luteinizing hormone, estradiol, and female age in women with recurrent miscarriage and to compare women with explained and idiopathic recurrent miscarriage.

**Design:**

Retrospective cohort study.

**Setting:**

University hospital, tertiary care center.

**Patients:**

Women with recurrent miscarriage (78 explained, 66 idiopathic).

**Intervention(s):**

None.

**Main Outcome Measures(s):**

Anti-Mullerian hormone, basal follicle stimulating hormone, luteinizing hormone, estradiol, and age.

**Results:**

Anti-Mullerian hormone and estradiol were significantly lower in women with idiopathic recurrent miscarriage (median 1.2 ng/ml, IQR 0.6–2.1, and median 36.5 pg/ml, IQR 25.8–47.3, respectively) than in women with explained recurrent miscarriage (median 2.0 ng/ml, IQR 1.1–2.7, and median 42.5 pg/ml, IQR 32.8–59.8, respectively; p<0.05). Optimized cut-off values for the prediction of idiopathic recurrent miscarriage were <39.5 pg/ml for estradiol (sensitivity: 63.3%, 95% CI: 50.9–75.1; specificity: 56.4%, 95% CI: 44.7–67.6) and <1.90 ng/ml for anti-Mullerian hormone (sensitivity: 72.7%, 95% CI: 60.4–83.0; specificity: 52.6%, 95% CI: 40.9–64.0).

**Conclusion:**

Idiopathic recurrent miscarriage was associated with lower basal estradiol and anti-Mullerian hormone levels compared to explained recurrent miscarriage.

## Introduction

The definition of recurrent miscarriage (RM) is based on three or more consecutive pregnancy losses, with the same partner, before the completed 20^th^ week of gestation [1]. Even after detailed evaluation of RM, in about 50% neither anomalies nor abnormal test results are found. These RM are labeled as unexplained or idiopathic (IRM) [2]. Very recently, the first study on anti-Mullerian hormone (AMH) levels in women with RM suggested that RM could be associated with diminished ovarian reserve [3]. This finding is reasonable, since the age-related decline in female fertility is frequently associated with an increased risk of miscarriage, including first trimester miscarriage [4]. In other words, there is an association between advanced biological maternal age and RM.

It has already been argued that ineffective management of couples with RM was caused by both under- and over-diagnostics, which could result in unnecessary tests and costs [5,6]. If diminished ovarian reserve is a major risk factor for IRM, unnecessary testing in affected women could be avoided. Thus, the aims of this study were (i) to evaluate well-known markers for ovarian reserve, e.g., AMH, basal follicle stimulating hormone (FSH), basal luteinizing hormone (LH), basal estradiol, and female age [4], in women with RM, (ii) to compare women with explained and idiopathic RM, and (iii) to test the above-mentioned factors as predictors for the presence of IRM. These aims could be achieved: We were able to demonstrate that IRM was associated with lower basal estradiol and AMH levels compared to explained recurrent miscarriage. However, the predictive value of these parameters for the presence of IRM was limited.

## Material and Methods

In a retrospective study, all 156 women with RM who had undergone a complete diagnostic evaluation at our department between January 2006 and January 2013 were included. All patients’ data included in this study are presented in S1 File. We excluded 12 women with polycystic ovary syndrome, since they would have inherently elevated AMH and LH-levels primarily because of their PCOS and not because of their ovarian reserve. This resulted in a population of 144. The study was approved by the Institutional Review Board of the Medical University of Vienna S2 File (IRB number 1098/2013). Data in this retrospective study was anonymized therefore waiving the need for consent. There was no funding for this study.

RM was defined based on a documented history of at least three spontaneous, consecutive miscarriages before 20 weeks’ gestation, with the same partner. All women had undergone a standard diagnostic evaluation. This included diagnostic hysteroscopy to exclude intrauterine synechia and uterine malformations; thrombophilia screening including protein S antigen, protein C activity, aPC-resistance, and antithrombin III activity; paternal and maternal karyotype; cervical cultures for chlamydia, ureaplasma, and mycoplasma; a comprehensive hormonal status including thyroid-stimulating hormone (TSH) and antibodies against thyroid peroxidase (TPO-Ab) and thyroglobulin (Tg-Ab); evaluation of diabetes mellitus with HbA1c assessment; and evaluation of antiphospholipid syndrome with IgM and IgG anti-cardiolipin antibody assessment (normal ranges: <10 IU/ml and <7 IU/ml, respectively) and IgM and IgG anti-beta-2-glycoprotein I antibody assessment (normal ranges for both parameters: <5 IU/ml) [7]. Moreover, basal serum levels of TSH, prolactin, FSH, LH, AMH, testosterone, androstenedione, dehydroepiandrosterone-sulfate, and 17-hydroxy-progesterone were also measured routinely. For this analysis, IRM was defined if no abnormalities had been found using these tools, including polycystic ovary syndrome defined by the revised criteria of the European Society of Human Reproduction and Embryology and the American Society for Reproductive Medicine [8], adrenogenital syndrome, hyperprolactinemia, and TSH levels >2.5 IU/ml.

Data were retrieved by retrospective chart review. Blood samples were obtained from a peripheral vein on the 3^rd^ to the 5^th^ day of a menstrual cycle. All examined serum parameters were determined in the central laboratory of the Vienna General Hospital, Austria, using commercially available assays. Radioimmunoassays were used to determine serum levels of estradiol (Autodelfia; Wallac Oy, Turku, Finland), LH (Autodelfia; Wallac Oy, Turku, Finland), FSH (Enzymun ES700; Boehringer Mannheim, Mannheim, Germany), and anti-Mullerian hormone (AMH; DSL Active MIS/AMH assay; Beckman Coulter Inc., Brea, USA).

Nominal variables are reported as numbers and frequencies, and continuous variables with median and range. Statistical analyses were performed with the SPSS software package, version 17 (SPSS, Chicago). Differences between groups were tested using the Fisher’s exact test for nominal variables and analysis of variance (ANOVA) for numeric variables. A logistic regression model was used to test the statistical significance of all coefficients. P-values of the likelihood ratio test, and ß-values with standard deviations are given, as well as Nagelkerke’s R^2^ for the goodness of fit of the whole multivariate model. Receiver Operating Characteristic (ROC) curves were computed to test a numeric variable’s predictive value. For these analyses, the area under the curve with standard error and 95% confidence intervals (95% CI) are provided. Optimized cut-off values were calculated as the value that reached the highest sum of sensitivity plus specifity according to the results of the ROC. Differences were considered significant if *p<* 0.05.

## Results

During the complete diagnostic evaluation, factors associated with RM were found in 78 women (Table 1), whereas 66 women were diagnosed with IRM. As stated above, 12 women were excluded for polycystic ovary syndrome. Notably, these women revealed significantly higher AMH levels than women with RM (median 7.6ng/ml, IQR 6.8–8.8 vs. median 1.6 ng/ml, IQR 0.8–2.6; *p<* 0.001). Neither the median number of previous miscarriages (3, IQR 3–4 vs. 3, IQR 3–4; respectively; *p =* 0.900) nor the median body mass index (24.3 kg/m^2^, IQR 21.0–30.0 vs. 25.4 kg/m^2^, IQR 21.3–30.0; respectively; *p =* 0.937) differed significantly between women with explained RM and IRM.

**Table 1:** Results of complete diagnostic evaluation in women with explained RM (*n =* 78).

Uterine abnormalities (malformations, Asherman syndrome)	28 (35.9)
Thrombophilic defects	10 (12.8)
Antiphospholipid syndrome	12 (15.4)
Parental chromosomal abnormalities	10 (12.8)
Hypothyroidism	17 (21.8)
Hyperthyroidism	8 (10.3)
Diabetes mellitus	9 (11.5)

Due to multiple citations the factors sum up to more than 100%. Data are presented as n (%).

**Fig 1:**
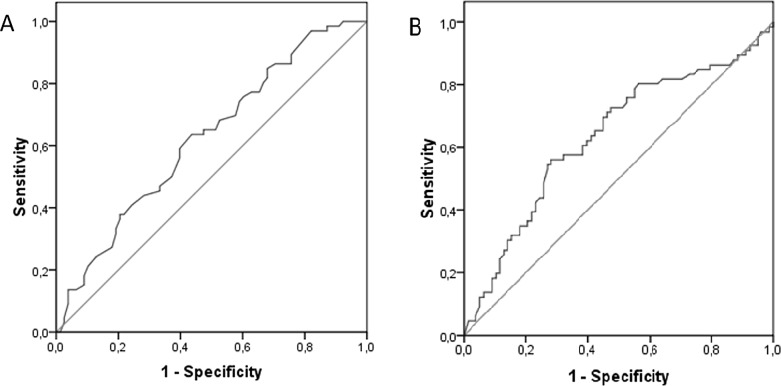
ROC curves for the prediction of idiopathic recurrent miscarriage based on estradiol and AMH levels. (A) Estradiol level. (B) AMH level.

Details on ovarian reserve parameters are provided in Table 2. Only AMH and estradiol differed between the groups (*p<* 0.05), with women who had IRM revealing significantly lower levels, whereas female age, FSH, and LH did not differ (*p>* 0.05). A Nagelkerke’s R^2^ of 0.155 indicated that this model explained 15.5% of the variability. We calculated ROC curves (Fig 1) for the significant predictors (AMH: AUC = 0.634, standard error = 0.047; 95% CI: 0.542–0.726; estradiol: AUC = 0.624; standard error = 0.046; 95% CI: 0.533–0.714). This resulted in the following optimized cut-off levels for the prediction of IRM: estradiol <39.5 pg/ml (sensitivity: 63.3%, 95% CI: 50.9–75.1; specifity: 56.4%, 95% CI: 44.7–67.6) and AMH <1.90 ng/ml (sensitivity: 72.7%, 95% CI: 60.4–83.0; specifity: 52.6%, 95% CI: 40.9–64.0).

**Table 2:** Uni- and multivariate binary logistic regression models for the prediction of idiopathic recurrent miscarriage.

			Univariate analysis		Multivariate analysis	
Parameter	Explained RM	Idiopathic RM	ß	p	ß	p
	(n = 78)	(n = 66)	(standard deviation)		(standard deviation)	
Age (years)	33 (28;36)	33 (28;38)	0.004 (0.027)	0.896	-0.062 (0.040)	0.124
LH (IU/l)	6.4 (4.5;9.3)	6.5 (4.6;11.0)	-0.002 (0.012)	0.880	-0.017 (0.018)	0.355
FSH (IU/l)	5.1 (3.7;7.4)	3.7 (5.6;8.3)	0.035 (0.026)	0.180	0.036 (0.036)	0.320
Estradiol (pg/ml)	42.5 (32.8;59.8)	36.5 (25.8;47.3)	-0.027 (0.010)	0.005	-0.027 (0.010)	0.007
AMH (ng/ml)	2.0 (1.1;2.7)	1.2 (0.6;2.1)	-0.322 (0.154)	0.037	-0.546 (0.235)	0.020

Data are presented as median (interquartile ranges).

## Discussion

This retrospective analysis suggests that IRM is associated with lower AMH and basal estradiol values than explained RM. Notably, this observed effect was valid, regardless of maternal age, which did not differ between the groups. This is in line with the recent observations of Atasever et al., who found decreased markers of ovarian reserve in women with RM, compared to age-matched fertile women [3]. Markers of ovarian reserve are quite comparable between this recent study and our data. When focusing on AMH, we report a slightly lower median level in our population with explained RM than Atasever et al. did in their general population (2.0 ng/ml vs. 2.9 ng/ml, respectively). However, women in our data set were a little older (33 vs. 29 years).

It has been reported that about 35%–75% of miscarriages are associated with chromosomal abnormalities of the embryo caused by decreased oocyte quality [9,10]. It seems reasonable that women with IRM are more likely to have diminished ovarian reserve, since decreased oocyte quality—with a, therefore, increased tendency toward embryonic chromosomal abnormalities—would not be detectable in the course of a standardized diagnostic evaluation for RM.

It has already been reported that cytogenetic analysis of abortion specimens from women with RM revealed a significantly higher percentage of chromosomally abnormal abortions than in women in the general population [11,12]. Accordingly, high rates of abnormal embryonic development and cytogenetic defects were found in women with RM, as evaluated by hystero-embryoscopy [13]. Moreover, diminished ovarian is even greater than the reliability of chronologic age [14].

In our multivariate model, only lower basal estradiol and AMH levels predicted the presence of IRM, whereas basal FSH and LH levels, as well as age, did not. This might put more emphasis on the value of AMH. The latter has been suggested to be more reliable than FSH, which might vary from cycle to cycle. The correlation between AMH and oocyte quality seems reassuring [15], although it has been debated controversially [16]. However, it should be mentioned that the explanatory power of our model is weak, with a Nagelkerke’s R^2^ of 0.155. This seems validated by the optimized cut-off values for estradiol and AMH, which lead to sums of sensitivity and specifity of only about 120%. Thus, it could be argued that only a minor percentage of IRM cases can be explained by diminished ovarian reserve.

In addition, significantly lower estradiol levels were found in women with IRM than in those with explained RM (Table 2). This is somehow in contrast to the findings of Atasever et al. who did not find differences in estradiol levels between women with and without RM [3]. Moreover, elevated basal estradiol levels are usually considered a sign of beginning diminished ovarian function. This can be seen indicative for hastened oocyte development [17]. One could hypothesize that women who are still able to conceive spontaneously but then tend to experience RM are in an earlier stage of losing their ovarian function. However, we find it hard to comment on our surprising finding.

The following study weaknesses have to be discussed: not all parameters of ovarian reserve could be included in our data set. Thus, data on antral follicular count, Inhibin B, and stimulation tests [4] are not available. Moreover, the small sample size and the retrospective design of the study must be considered as study limitations. It must also be mentioned that an unexpectedly high percentage of uterine abnormalities of about 35% was found. We find it hard to comment on this result. It might be due to the fact that there was some kind of selection bias, since some of the women with RM and easily detectable causes including thyroid disorders and polycystic ovary syndrome might have been diagnosed and treated by non-specialized gynecologists and, thus, have not been referred to our department.

Due to the retrospective study design, we cannot provide data on prothrombin, factor VIII and genetic factors of coagulation disorders. These have not been part of our screening program, since they had been discussed controversially in literature [18]. Moreover, one might argue that classifying RM as “idiopathic” without knowledge on fetal chromosomal testing was incorrect. It has been shown that in IRM, 54% could be related to aneuploidies [19]. However, in clinical routine, karyotyping of abortus is rarely used, and, thus, one often relies on parental karyotyping which has been performed in all of our cases. When parental chromosomal abnormalities were detected, cases were rated as “explained”. In doing this, we adhere to current recommendations concerning these definitions and diagnostic evaluation [18]. We agree that defining cases of RM that could have been associated with fetal chromosomal abnormalities as “idiopathic” is somehow incorrect. However, our findings seem to support the approach that fetal aneuploidies associated with advanced maternal biologic age are of influence for the occurrence of RM [3,20]. Hopefully, one will not define RM associated with low AMH levels and higher maternal age as “idiopathic” in the future, but these new insights might need to be implemented in future recommendations.

However, to the best of our knowledge, this is the first study to compare markers of ovarian reserve between women with explained RM and IRM, and it is the second study on AMH levels in women with RM. In conclusion, IRM was associated with lower basal estradiol and AMH levels, compared to explained RM. However, the predictive values of estradiol and AMH were considerably low, thus these parameters seem not eligible as independent diagnostic tools in this setting. Larger prospective trials are warranted to prove these findings and answer the question of whether IRM can reliably predict which women have a very low ovarian reserve, and, thus, the complex diagnostic evaluation could be omitted.

## Supporting Information

S1 File Dataset. Data of patients suffering from recurrent miscarriage. (XLSX)
(XLSX)

S2 File Ethic committee approval. (PDF)

S3 File STROBE Checklist. (PDF)
