# Supplementary material for: Decreased Ovarian Reserve Predicts Inexplicability of Recurrent Miscarriage? A Retrospective Analysis
Source: PLoS One. 2016 Sep 14;11(9):e0161606. doi: 10.1371/journal.pone.0161606 (PMC5023100; doi:10.1371/journal.pone.0161606)
Supplement: S2 File — (PDF) [file pone.0161606.s002.pdf]

ethik kommission
Medizinische Universität Wien

Borschkegasse 8b/6
A-1090 Wien, Austria

T +43 1 40 400 – 2147, 2244
F +43 1 40 400 – 1690
ethik-kom@meduniwien.ac.at
ethikkommission.meduniwien.ac.at

Votum:

EK Nr: 1098/2013
Projekttitel: Analyse der Risikofaktoren von Patientinnen mit Abortus habitualis im Patientenkollektiv des AKH Wien
Antragsteller/in: Frau Sophie Taborsky
Institution: Universitätsklinik für Frauenheilkunde
Sponsor: Medizinische Universität Wien

Teilnehmende Prüfzentren:

Ethik-Kommission	Prüfzentrum	Prüfärztin/arzt
Ethikkommission der Medizinischen Universität Wien	Universitätsklinik für Frauenheilkunde	Herr Priv. Doz. Dr. Johannes Ott

Die Stellungnahme der Ethik-Kommission erfolgt aufgrund folgender eingereichter Unterlagen:

Dokument	Name	Version	Datum
Sonstige	Verpflichtungserklärung	1	01.02.2013
Studienprotokoll (Prüfplan)	Projektplan V2 20.03.2013_hervorgehobene Veränderungen	Version 2	20.03.2013
	Projektplan V2 20.03.2013	Version 2	20.03.2013

Die Kommission fasst folgenden Beschluss (mit X markiert):

☒	Es besteht kein Einwand gegen die Durchführung der Studie.

Kommentare:
Zum Prüfplan:

Die Ethik-Kommission ersucht um Stellungnahme zu folgenden Punkten:

Im Abstract wird von einem prospektiven Teil der Studie (ein telefonisches Follow-up der Patientinnen) gesprochen. Ist ein Patientinnenkontakt geplant?
Protokoll Seite 6: "Interessant ist auch, ob genetische Unterschiede das Abortgeschehen beeinflussen, da im Patientinnenkollektiv viele verschiedene Ethnizitäten vertreten sind". Ist eine genetische Untersuchung vorgesehen?

Zum Antrag:
Es wird angeführt, dass im Rahmen der Qualitätssicherung der Abteilung für Gynäkologie und gynäkologische Endokrinologie die Schwangerschaftsrate und Baby-take-home Rate telefonisch von den Patientinnen erfragt werden.

Ist diese Kontaktaufnahme Teil der Routineversorgung und sind die Daten zum Zeitpunkt der Auswertung schon vorhanden, oder gibt es - wie im Studienprotokoll angeführt - eine prospektive Kontaktaufnahme im Rahmen der Studie?
Die Ethik-Kommission ersucht um Stellungnahme.

Zur Patienteninformation:
Falls die Studie nicht rein retrospektiv ist, sondern in Zukunft noch Daten aus der telefonischen Befragung von Patientinnen erhoben werden sollen, so ist eine Information für die Teilnehmerinnen vorzulegen.

Zur Versicherungsbestätigung: nicht erforderlich

Andere:
Das unterschriebene Antragsformular ist nachzureichen.

Die Ethik-Kommission ersucht die Antragsteller, bei der Wiedervorlage von geänderten Unterlagen ein Exemplar mit hervorgehobenen Änderungen beizulegen.

Ergänzende Kommentare:

Nachtrag vom 3. April 2013:
Die Antragsteller legen am 20.03.2013 überarbeitete Unterlagen vor, die von der Ethik-Kommission akzeptiert werden. Das unterzeichnete Antragsformular wurde nachgereicht.

Die Ethik-Kommision geht - rechtlich unverbindlich - davon aus, daß es sich **nicht** um eine klinische Prüfung gemäß AMG/MPG handelt.

Die aktuelle Mitgliederliste der Ethik-Kommission ist unter der Adresse http://ethikkommission.meduniwien.ac.at/ethik-kommission/mitglieder/ abrufbar. Mitglieder der Ethik-Kommission, die für diesen Tagesordnungspunkt als befangen anzusehen waren und daher laut Geschäftsordnung an der Entscheidungsfindung/Abstimmung nicht teilgenommen haben: Frau Christine Kurz

ACHTUNG: Unter Berücksichtigung der "ICH-Guideline for Good Clinical Practice" gilt dieser Beschluss **ein Jahr ab Datum der Ausstellung**. Gegebenenfalls hat der Antragsteller eine Verlängerung der Gültigkeit rechtzeitig zu beantragen.

Dieses Votum ist für berechtigte Benutzer/innen in digitaler Form unter der Adresse https://ekmeduniwien.at/vote/2204/download/ abrufbar.

**Signaturwert**	v1+HJymA671HCfQnxb/xjQ+0/CgZoE0STYXTkoXC7706kevqNzixINql//JKdoXk8aNGwrHBtIdowVQ/gIx5RQ==	
	**Unterzeichner**	Dr. Ernst Singer
	**Aussteller-Zertifikat**	CN=a-sign-Premium-Sig-02,OU=a-sign-Premium-Sig-02,O=A-Trust Ges. f. Sicherheitssysteme im elektr. Datenverkehr GmbH,C=AT
	**Serien-Nr.**	456499
	**Methode**	urn:pdfsigfilter:bka.gv.at:binaer:v1.1.0
	**Parameter**	etsi-moc-1.1@cda3e3ae
**Prüfinformation**	Informationen zur Prüfung der elektronischen Signatur und des Ausdrucks finden Sie unter: http://www.signaturpruefung.gv.at	
**Datum/Zeit-UTC**	2013-04-03T16:57:19Z	
